# Human Skin Culture as an *Ex Vivo* Model for Assessing the Fibrotic Effects of Insulin-Like Growth Factor Binding Proteins 

**DOI:** 10.2174/1874312900802010017

**Published:** 2008-03-28

**Authors:** Hidekata Yasuoka, Adriana T. Larregina, Yukie Yamaguchi, Carol A. Feghali-Bostwick

**Affiliations:** Department of 1 Medicine; Department of 2 Dermatology, 2 Immunology and; Department of 3 Pathology, University of Pittsburgh, Pittsburgh, Pennsylvania, USA

## Abstract

Systemic sclerosis (SSc) is a connective tissue disease of unknown etiology. A hallmark of SSc is fibrosis of the skin and internal organs. We recently demonstrated increased expression of IGFBP-3 and IGFBP-5 in primary cultures of fibroblasts from the skin of patients with SSc. *In vitro,* IGFBP-3 and IGFBP-5 induced a fibrotic phenotype and IGFBP-5 triggered dermal fibrosis in mice. To assess the ability of IGFBPs to trigger fibrosis, we used an *ex vivo* human skin organ culture model. Our findings demonstrate that IGFBP-3 and IGFBP-5, but not IGFBP-4, increase dermal and collagen bundle thickness in human skin explants, resulting in substantial dermal fibrosis and thickening. These fibrotic effects were sustained for at least two weeks. Our findings demonstrate that human skin *ex vivo* is an appropriate model to assess the effects of fibrosis-inducing factors such as IGFBPs, and for evaluating the efficacy of inhibitors/therapies to halt the progression of fibrosis and potentially reverse it.

## INTRODUCTION

Systemic sclerosis (SSc) is a connective tissue disease whose hallmarks include fibrosis, immune dysregulation, and vascular abnormalities. Dermal fibrosis is a prominent feature of SSc. The most commonly used mouse models of SSc are those arising from genetic mutations or treatment with bleomycin [1]. Although these murine models have proven to be useful for understanding the role of various factors in fibrosis, the ultimate goal of our research is to determine the applicability of our findings in humans who are afflicted with the disease. Furthermore, comparative studies have revealed important anatomical cellular and functional differences between human and mouse skin [2].

Using human skin as an *ex vivo* organ model of fibrosis has several advantages. First, the skin explants remain viable in culture for at least two weeks. Second, factors and vectors can be readily administered to skin explants *via *deep and/or superficial injections to affect both the epidermis and dermis or mainly the dermis as these epidermal-dermal explants consist of both epidermis and dermis. Furthermore, skin explants contain all cell types resident in the skin which are capable of exerting their physiological effects on the tissue and neighboring cells. Skin explants have been successfully used to express transgenes in both the epidermal and dermal layers for vaccination and immunotherapeutic purposes [3-6].

Insulin-like growth factor binding proteins (IGFBPs) are a family of six proteins identified for their ability to bind IGF-I with high affinity [7, 8]. We have previously shown that IGFBP-5 is over-expressed in fibrotic lung and skin tissues in patients with SSc and in lung tissues of patients with idiopathic pulmonary fibrosis [9-1]. We have also demonstrated that IGFBP-5 can induce the development of a fibrotic phenotype *in vitro* in primary fibroblasts and can trigger dermal and pulmonary fibrosis *in vivo* in murine models [11, 12]. Since our original observation of increased IGFBP-5 levels was made in human samples and the final goal of our studies is the applicability of our findings in humans, we optimized use of an ‘*ex vivo*’ organ culture model using human skin. The effect of IGFBP-5 on the development of a fibrotic phenotype was examined in this *ex vivo* skin explant model. Our findings suggest that expressing IGFBP-5, and to a lesser extent IGFBP-3, in normal skin explants results in increased dermal thickness and increased collagen bundle thickness, thus recapitulating the dermal fibrosis seen in patients with SSc.

## MATERIALS AND METHODOLOGY

**Human Samples: **Human skin was obtained from corrective plastic surgery. All tissues were obtained according to the guidelines of the University of Pittsburgh and under a protocol approved by the Institutional Review Board of the University of Pittsburgh. Subcutaneous fat tissue was removed and skin tissue was cut into 1.5 cm x 1.5 cm sections. Adenoviral (Ad) constructs were injected intradermally in a volume of 100 µl 1x PBS. Explants containing complete epidermal and dermal layers were cultured in an air liquid interface with the epidermal and keratin layers side up and exposed to air. The culture medium was replaced daily and consisted of Dulbecco’s modified Eagle’s medium (DMEM) (Mediatech, Herndon, VA) supplemented with 10% FBS (Sigma-Aldrich, St Louis, MO), penicillin, streptomycin, and anti-mycotic agent (Invitrogen Life Technologies, Carlsbad, CA). At the indicated time points, skin tissue was harvested and fixed in 10% formalin prior to embedding in paraffin. Skin punch biopsies were obtained from the clinically affected and unaffected skin of patients with SSc as we have previously described [9, 11].

**Adenoviral Constructs: **Replication deficient adenoviruses serotype 5 encoding human IGFBP-3, IGFBP-4, or IGFBP-5 were generated as previously described [10]. Adenovirus serotype 5 lacking cDNA was used as a control. Adenoviruses (1 x 10^8^ pfu) were injected intradermally in a 100 µl volume.

**Immunohistochemistry (IHC): **Six µm sections of paraffin embedded tissues were deparaffinized and endogenous peroxidases were quenched with 3% H_2_O_2_. Sections were blocked with 5% serum and incubated with polyclonal anti-IGFBP-5 antibody (Gropep Ltd, Adelaide, Australia) or IgG control antibody (Lab Vision Corporation, Fremont, CA). Sections were washed and incubated with biontinylated secondary antibody (Vector Laboratories, Burlingame CA). Bound secondary antibody was detected using the Vectastain ABC kit (Vector Laboratories) and Zymed AEC Red kit (Zymed, San Francisco CA). A light hematoxylin counterstain was used to identify nuclei using Hematoxylin QS (Vector Laboratories). Images were taken on a Nikon Eclipse 800 microscope (Nikon Instruments Inc., Huntley, IL) using identical camera settings.

**Measurement of Skin Dermal and Collagen Bundle Thickness:**Six µm sections of paraffin-embedded skin tissue were stained with hematoxylin and eosin (H &amp; E). Images were taken on a Nikon Eclipse 800 microscope. The thickness of the dermis and of individual collagen bundles was measured using Microsuite™ Software (Olympus America Inc.) as we previously described [11]. Thickness was measured in 5 random fields in each sample. Data are shown in arbitrary units.

**Statistical Analysis:**Dermal and collagen bundle thickness were analyzed using the Mann-Whitney *U* test.

## RESULTS

### Increased IGFBP-5 Expression in Skin Tissues of Patients with Systemic Sclerosis

We had previously shown that primary fibroblasts from skin punch biopsies of patients with SSc express significantly greater IGFBP-5 than healthy donor fibroblasts [9]. Furthermore, fibroblasts from the clinically affected skin of patients with SSc secrete and deposit into their extracellular matrix significantly more IGFBP-5 compared to fibroblasts from the patients’ clinically unaffected skin [11]. To determine whether increased IGFBP-5 expression *in vitro* parallels expression *in vivo*, IGFBP-5 levels in skin tissue from the clinically affected and unaffected skin of two SSc patients and those of two healthy controls were examined by IHC. Fig. (**1**) shows that IGFBP-5 protein levels are noticeably increased in SSc affected skin compared to SSc unaffected and healthy donor skin. IGFBP-5 was mainly expressed in epithelial cells, dermal fibroblasts and endothelial cells. These results extend our previous findings on increased IGFBP-5 and demonstrate that the increased production of IGFBP-5 *in vitro* by SSc fibroblasts reflects the aberrant expression *in vivo*.

### Expression of IGFBP-5 in Human Skin Explants Parallels Expression and Distribution of the Protein in SSc Skin

Human skin explants were injected intradermally with control adenovirus (cAd), or adenovirus expressing IGFBP-5 (Ad5). One and two weeks post-injection, skin explants were fixed and embedded in paraffin. Histological examination of sections stained with hematoxylin and eosin revealed no morphological changes by at least two weeks. Electron microscopy confirmed these findings (data not shown). We previously reported that IGFBP-5 is increased in dermal fibroblasts from patients with SSc [9, 11]. To determine whether adenovirally encoded IGFBP-5 results in expression levels comparable to those observed in SSc skin, Ad-treated human skin explants and SSc skin sections were examined by IHC using anti-IGFBP-5 antibody. Fig. (**2**) shows that adenoviral expression of IGFBP-5 in human skin explants *ex vivo* results in expression intensity and patterns comparable to those observed in SSc patient skin shown in Fig. (**1**).

### Efficient Expression of IGFBP-5 in Human Skin Explants Results in Increased Dermal Thickness

**Fig. (3):**
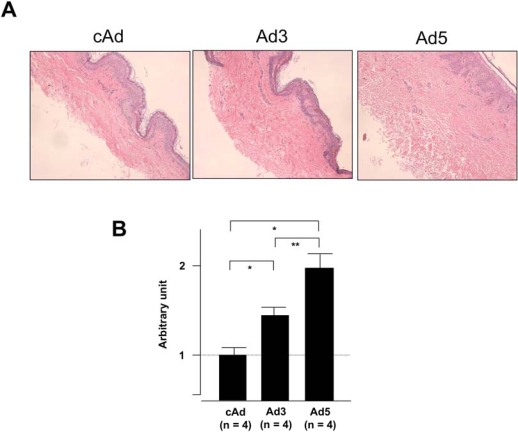
IGFBP-5 expression increases dermal thickness. Human skin explants were injected with control Ad (cAd), Ad expressing IGFBP-3 (Ad3) or Ad expressing IGFBP-5 (Ad5). Dermal thickness was assessed one week post-injection. **(A)** Expression of IGFBP-5 and to a lesser extent IGFBP-3 results in increased dermal thickness. H&E, 40x. **(B)** Graphical presentation of dermal thickness. *p < 0.02; **p < 0.04. Data represent four independent experiments using human skin explants from four donors.

**Fig. (4):**
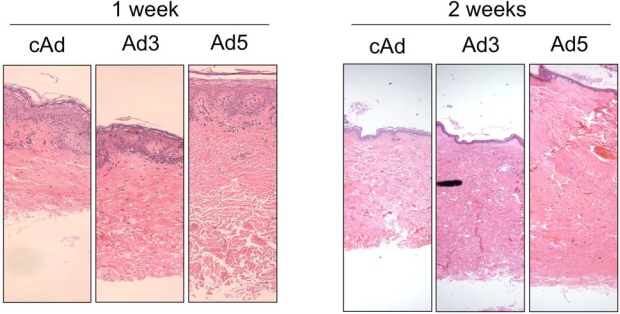
IGFBP-5, and to a lesser extent, IGFBP-3, increase dermal thickness one and two weeks post injection. Human skin explants were injected with cAd, Ad3 or Ad5. H & E, 40X.

To determine whether IGFBP-5 exerts pro-fibrotic effects *ex vivo* in human skin, skin tissues from four different donors were injected intradermally with Ad expressing IGFBP-3 (Ad3), IGFBP-5 (Ad5) or no cDNA (cAd). Skin tissue was harvested one week post adenoviral administration and sections were stained with hematoxylin and eosin. Fig. (**3A**) shows increased dermal thickness of human skin expressing IGFBP-5, whereas IGFBP-3 induced modest thickening of the skin. The increase in dermal thickness induced by IGFBP-3 and IGFBP-5 was statistically significant (Fig. **3B**). Data shown are representative of four independent experiments. Furthermore, IGFBP-3 and IGFBP-5 induced increase in dermal thickness is maintained for two weeks (Fig. **4**). Two weeks post adenoviral injection, IGFBP-5 expression resulted in deposition of dermal collagen that was uniformly stained with eosin (Fig. **4**).

### Increased Expression of IGFBP-5 in Human Skin Explants Results in Increased Thickness of Individual Collagen Bundles

In addition to measuring dermal thickness, we assessed the effect of IGFBP-3 and IGFBP-5 expression on the thickness of individual collagen bundles in the dermal layer. IGFBP-3 and IGFBP-5 expression resulted in significantly increased collagen bundle thickness and IGFBP-5 resulted in strong staining of collagen bundles with eosin as shown in

Fig. (**5**). The increase in dermal and collagen bundle thickness by the pattern of eosin staining were similar if not identical to those changes observed in the skin of patients with SSc.

### Increased Expression of IGFBP-4 Does Not Alter Dermal or Collagen Bundle Thickness

Our previous findings demonstrated that both IGFBP-3 and IGFBP-5 trigger a fibrotic phenotype *in vitro* when applied to primary human fibroblasts [10]. *In vivo*, IGFBP-5 but not IGFBP-3, induced dermal fibrosis in mice [11]. The differential effects of IGFBP-3 in human fibroblasts versus mouse skin may be due to the fact that human rather than mouse IGFBP-3 was expressed adenovirally, and IGFBP-3 may exert species-specific effects on the matrix. To determine if the induction of fibrosis is a general effect of IGFBPs or a function specific to IGFBP-3 and IGFBP-5, the assays were repeated with skin samples injected with cAd, Ad3, Ad5, and an adenovirus expressing IGFBP-4 (Ad4) as a related protein control. Fig. (**6**)****shows that IGFBP-4, unlike IGFBP-3 and IGFBP-5, does not modulate dermal or collagen bundle thickness of human skin explants.

## DISCUSSION

Studies have shown that percutaneous permeation of molecules differs in human and rodent skin [13]. Human skin from cosmetic surgery and amputations has been used to assess the transdermal absorption of molecules and its permeation and exerts similar properties to those of pig skin whereas permeation rates of rodent skin are much greater [13]. Human skin explants have been in use as an *ex vivo* organ culture model for a few different applications including the expression of DNA [14], the application of inhibitors to metalloproteinases and other proteinases [15], and treatment with cytokines and growth factors such as IFN-γ [16].

Recently, the use of human skin explants for evaluating the effect of potential therapeutic agents has been reported. In an attempt to interfere with the fibrotic process, Nath *et al.* recently reported using antisense oligonucleotides to reduce Collagen Type I expression *ex vivo* in a skin organ culture model that was maintained for 7 days [17]. To extend the application of human skin explants to *in vivo* gene therapy, Lippin *et al. *obtained skin explants from patients with chronic renal failure and anemia, injected them with an adenovirus expressing human erythropoietin, and reimplanted them into the patients [18]. Increased circulating erythropoietin levels were observed for 14 days and correlated with a rise in reticulocyte count. Although anti-erythropoietin antibodies were detected 3 months following implantation, this approach suggests that use of an *ex vivo* skin organ culture model can be extended to the clinical setting in diseases amenable to gene therapy.

Although numerous reports have focused on the therapeutic applications of human skin *ex vivo*, our findings suggest that a) human skin explants are valuable as a model to assess the effects of fibrotic factors and b) expression of factors such as IGFBP-5, which trigger a fibrotic phenotype *in vitro* in primary human fibroblasts and *in vivo* in mouse skin and lung tissues [10-12], induces fibrosis *ex vivo* in a skin organ culture model for at least two weeks. Use of skin explants obviates the need for rodent models and renders all findings directly applicable to human diseases such as SSc.

Our findings demonstrate that a) IGFBP-4 does not exert pro-fibrotic effects in human skin, and b) that IGFBP-3 and IGFBP-5 are pro-fibrotic *ex vivo*, although the effects of IGFBP-3 are more modest than those of IGFBP-5 suggesting that IGFBP-5 is a potent pro-fibrotic factor. Thus, IGFBP-3, -4, and –5 exert different effects in the human skin organ culture model. Both IGFBP-3 and IGFBP-5 induce dermal fibrosis with IGFBP-5 exerting a more drastic phenotype than IGFBP-3. In contrast, IGFBP-4 expression did not alter dermal or collagen bundle thickness. These three IGFBPs share features that are common to the IGFBP family of proteins: they have conserved N-terminal and C-terminal domains that contain 12 and 6 conserved cysteine residues, respectively. They bind IGF-I with high affinity and modulate its action, either potentiating or inhibiting it [7, 8]. IGFBPs also exhibit important differences. They can exert both IGF-I-dependent and –independent effects and differentially regulate cellular functions such as apoptosis of breast epithelial cells [19]. IGFBP-3 and IGFBP-5 are more structurally similar to each other than to IGFBP-4, a smaller IGFBP that has been shown to inhibit IGF actions [20]. Such differences may be responsible for the different effects of IGFBP’s in the development of dermal fibrosis.

We have previously shown that both IGFBP-3 and IGFBP-5 induce a fibrotic phenotype *in vitro* defined as increased collagen and fibronectin production and deposition in the ECM of primary human fibroblasts [10]. However, overexpression of human IGFBP-3 in mouse skin, unlike IGFBP-5, did not induce fibrosis [11]. In view of our current findings that both IGFBP-3 and IGFBP-5 induce fibrosis of human skin, it is likely that the inability of human IGFBP-3 to induce fibrosis in rodent skin is due to species-specific activities. Since IGFBP-5 is the most conserved of the IGFBPs, human IGFBP-5 was able to exert similar effects in both human and mouse skin. It is also conceivable that in our human skin explant model, fibrosis in response to IGFBP-3 and IGFBP-5 develops if circulating molecules that can counteract the effects of these IGFBPs are not available. Future directions will address this possibility by maintaining the explants in the presence and absence of autologous serum and or peripheral blood mononuclear cells.

Ideally, once fibrosis in response to IGFBP-5 or other factors is established *ex vivo*, the explants can be treated with agents specific to the fibrotic factors of interest for a targeted therapeutic approach. Thus, the *ex vivo* human skin model is valuable for assessing the fibrotic activity of molecules of interest as well as evaluating the efficacy of anti-fibrotic therapies in a milieu that renders the findings directly applicable to human disease.

## CONCLUSION

In conclusion, our findings demonstrate that skin explants can serve as a suitable *ex vivo* organ model to assess the role of IGFBP-3 and IGFBP-5 in the development of dermal fibrosis. Adenoviral expression of IGFBP-5 administered intradermally results in IGFBP-5 expression levels and distribution comparable to those seen in SSc patient skin. Furthermore, IGFBP-5, and to a lesser extent IGFBP-3, trigger fibrosis *ex vivo* in a human skin organ culture model. Thus, human skin explants are a suitable model to address the role of pathogenic molecules in the development of fibrosis and to assess the role of these molecules and potentially therapeutic factors in human disease.

## Figures and Tables

**Fig. (1):**
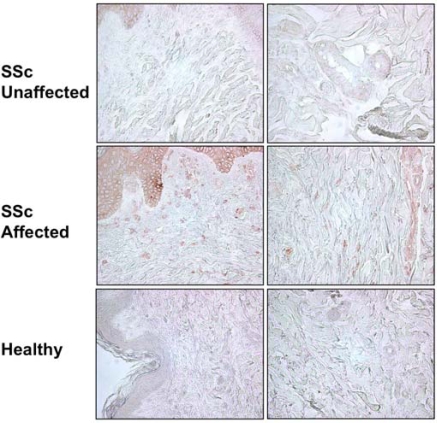
Increased IGFBP-5 expression in affected SSc skin. IGFBP-5 levels in skin tissues from the affected and unaffected skin of a patient with SSc and a healthy donor were examined using IHC. Immunoperoxidase, 400x.

**Fig. (2):**
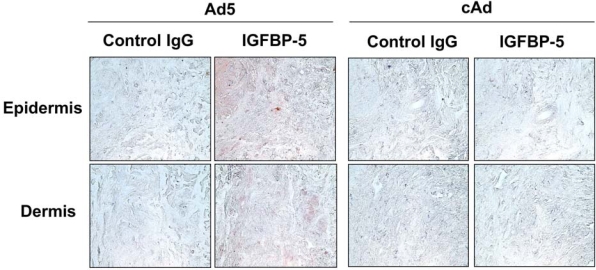
IGFBP-5 is detected in human skin explants injected intradermally with Ad expressing IGFBP-5. IGFBP-5 expression was examined by IHC one week post-adenoviral administration. Immunoperoxidase, 400x.

**Fig. (5):**
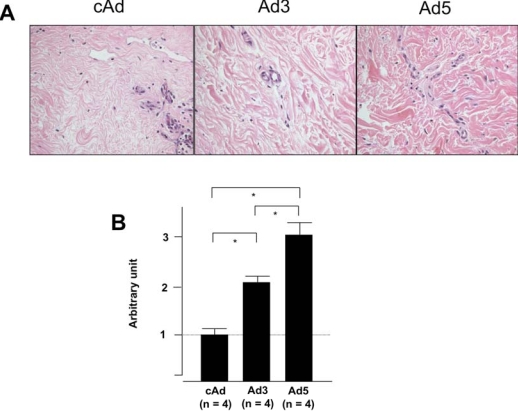
IGFBP-5 and IGFBP-3 expression increases collagen bundle thickness. Human skin explants were treated as in figure 3. The thickness of individual collagen bundles was measured. **(A)** IGFBP-3 and IGFBP-5 expression results in increased thickness of individual collagen bundles. **(B)** Graphical presentation of data presented in A. *p < 0.02. Data represent four independent experiments using skin explants from four different donors. H & E, 800X.

**Fig. (6):**
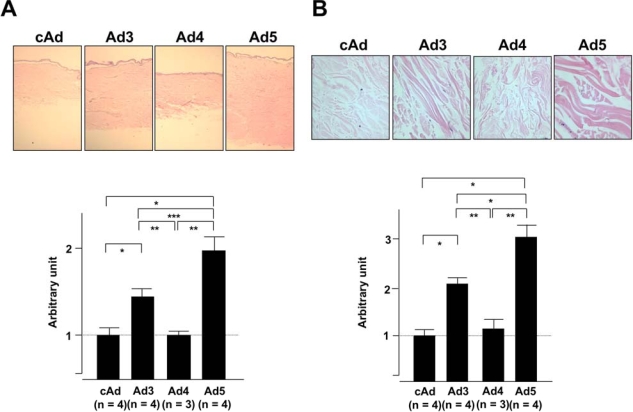
IGFBP-4 does not alter the thickness of the dermis or the collagen bundles. Human skin explants were treated as in Fig. (3) except that skin explants were injected with control cAd, Ad3, Ad5, or Ad expressing IGFBP-4 (Ad4). **(A)** Dermal thickness was measured in skin tissues one week post-injection. **(B)** Collagen bundle thickness was measured in skin tissues one week post-injection. *p < 0.02; **p < 0.03 ***p< 0.04. Panel A: H & E, 20X; Panel B: H & E, 800X.
